# Cytotoxic Natural Products Isolated from *Cryptogramma crispa* (L.) R. Br.

**DOI:** 10.3390/molecules28237723

**Published:** 2023-11-23

**Authors:** Andrea Estefania Carpinteyro Diaz, Lars Herfindal, Heidi Lie Andersen, Torgils Fossen

**Affiliations:** 1 Department of Chemistry and Centre for Pharmacy, University of Bergen, N-5007 Bergen, Norway; andrea.diaz@uib.no; 2 Department of Clinical Science and Centre for Pharmacy, University of Bergen, N-5009 Bergen, Norway; lars.herfindal@uib.no; 3 University Gardens, University of Bergen, Allégt. 41, N-5007 Bergen, Norway; heidi.andersen@uib.no

**Keywords:** *Cryptogramma crispa*, aerial parts, pterosin, pteroside, NMR, cytotoxicity

## Abstract

Parsley fern, *Cryptogramma crispa*, is a common fern in arctic–alpine regions, and even though this species has been known since ancient times and has been presumed to cause the poisoning of horses, its natural products have not previously been investigated. Here, we characterise 15 natural products isolated from the aerial parts of *Cryptogramma crispa*, including the previously undescribed compound 3-malonyl pteroside D. The structure determinations were based on several advanced 1D and 2D NMR spectroscopic techniques, Circular Dichroism spectroscopy and high-resolution mass spectrometry. The pteroside derivatives exhibited selective moderate cytotoxic activity against the acute myeloid leukaemia MOLM13 cell line and no cytotoxicity against the normal heart and kidney cell lines, suggesting that their potential anticancer effect should be further investigated.

## 1. Introduction

**Figure 1:**
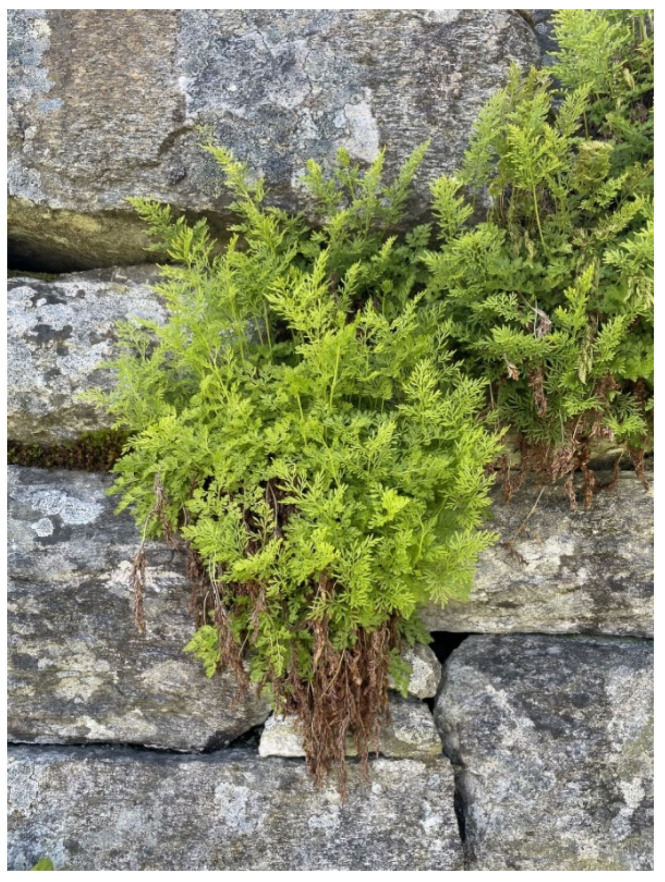
Aerial parts of *Cryptogramma crispa*. Photo: Andrea Estefania Carpinteyro Diaz.

The leptosporangiate fern genus *Cryptogramma* of the family Pteridaceae comprise nine species and is referred to as parsley fern, as its foliage resembles that of parsley [1]. *Cryptogramma crispa* (L.) R. Br. (Figure 1) is a small rupestral fern widely distributed from the montane to the subalpine zones of the temperate and boreal regions of Europe, with two main distribution centres: the northern area, including the highest mountains in the British Isles and west Scandinavia, and the southern area comprising the highest mountains in south Europe. Within its northern distribution area, *C. crispa* occurs over a broad altitudinal range, between 20 m and 2800 m above sea level [2,3].

*C. crispa* is a strongly calcifuge species that only grows on non-carbonaceous bedrocks, often forming patches. This species is quite hardy and can be covered by snow during the winter and receive lots of solar radiation during the summer [3,4].

According to observations within Norwegian traditional medicine, horses that excessively consume parsley ferns are susceptible to colic, thus explaining its Norwegian name hestespreng (which means “horse bloating”). Even though the traditional observation has not been unambiguously confirmed in modern studies [5], this species appears to be a potentially interesting source of bioactive natural products. While the existing literature regarding *C. crispa* is mainly limited to studies of the botanical characteristics of the plant, only a restricted number of studies have explored its phytochemical composition.

In the current literature, only relatively volatile compounds, including alkyl esters, fatty acids, primary alcohols, aldehydes, and alkanes, have hitherto been identified by GCMS from *C. crispa* [6]. Some carotenoid derivatives and shikimic acid derivatives have also been suggested to be present in this plant [7]. However, the structures of these compounds remain elusive. To the best of our knowledge, no studies exist in the current literature about the isolation and identification of pure compounds from this plant species.

As part of our ongoing research on the description of new natural products with potential applications as lead compounds of future anticancer drugs, we report on the characterisation of 15 natural compounds from the aerial parts of *C. crispa*, which have been identified from this plant species for the first time, including a novel compound, as well as their cytotoxic activity towards leukaemia cells and normal kidney and heart cell lines.

## 2. Results and Discussion

The methanolic extract obtained from the aerial parts from *Cryptogramma crispa* was concentrated under reduced pressure and subjected to separation via liquid/liquid partition with petroleum ether followed by ethyl acetate. The components of the aqueous and ethyl acetate phases were further separated via adsorption chromatography, gel filtration chromatography, and preparative HPLC.

**Figure 2:**
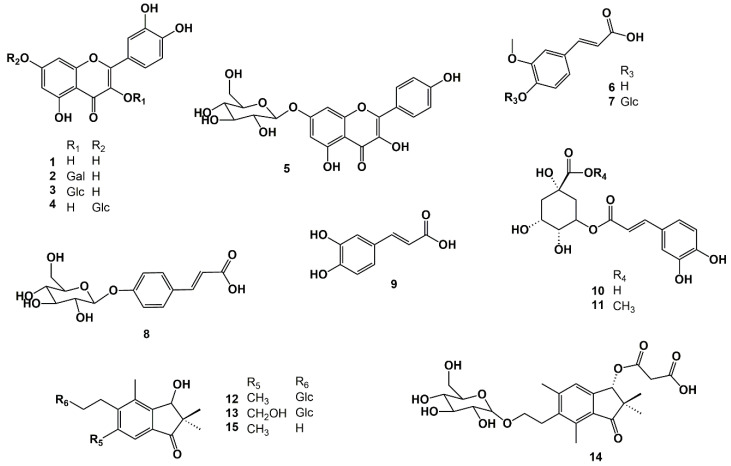
Molecular structures of compound **1**–**15** characterised from *Cryptogramma crispa* (L.) R. Br.

The fourteen known compounds isolated from *C. crispa* are: quercetin (**1**), quercetin 3-*O*-*β*-galactopyranoside (**2**), quercetin 3-*O*-*β*-glucopyranoside (**3**), quercetin 7-*O*-*β*-glucopyranoside (**4**), kaempferol 7-*O*-*β*-glucopyranoside (**5**), ferulic acid (**6**), ferulic acid 4-*O*-*β*-glucopyranoside (**7**), *p*-coumaric acid-4-*O*-*β*-glucopyranoside (**8**), caffeic acid (**9**), chlorogenic acid (**10**), chlorogenic acid methyl ester (**11**), pteroside D (**12**), pteroside X (**13**), and pterosin D (**15**) (Figure 2). These compounds were identified for the first time in parsley fern with using several 1D and 2D NMR spectroscopic techniques. Compound **13** was previously identified as pteroside X by Murakami et al. [8] from *Pteris fauriei* Hieron using 1D ^1^H NMR. In this paper, complete ^1^H and ^13^C data for pteroside X are available for the first time.

**Figure 3:**
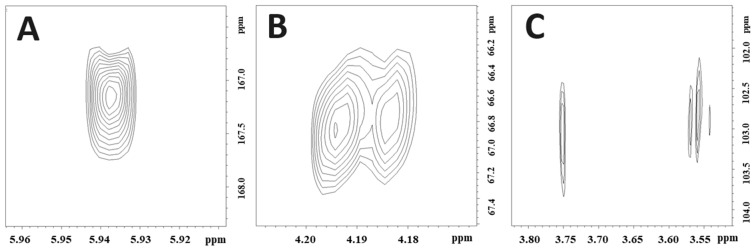
Important HMBC cross-peaks for structure determination of 3(R)-O-malonyl pteroside D (compound **14**). (**A**) The cross-peak at δ 5.94/167.2 (H-4/C-1″) confirms the malonyl substituent’s connection to the 1-indanon core. (**B**) The cross-peak at δ 4.18/66.8 (H-1′/C-14) and (**C**) the cross-peaks detected at δ 3.75/102.9 (H-14A/C-1′) and δ 3.56/102.9 /H-14B/C-1′), confirming the connection of the glucosyl unit to the alkyl group at C-14.

The UV spectrum of compound **14** recorded online during HPLC analysis exhibited λ_max_ values at 260, 215, and 198 nm (Figure S10), which are consistent with a 1-indanone derivative. The 1D ^1^H NMR spectrum and the 1D ^13^C NMR spectrum of **14** (Figures S1 and S2) showed the presence of an extensively substituted 1-indanone derivative, with only one aromatic hydrogen observed at ẟ 7.28 s (H-4) (Table 1) and one aliphatic hydrogen (H-3) observed at ẟ 5.94 s directly attached to the 1-indanone ring system. The 1D ^1^H NMR spectrum and the 1D ^13^C NMR spectrum of **14** showed the presence of four methyl groups at ẟ 1.19 (H-10), ẟ 0.96 (H-11), ẟ 2.42 (H-12), and ẟ 2.61 (H-15), respectively. The cross-peaks at ẟ 1.19/49.8 (H-10/C-2) and ẟ 0.96/49.8 (H-10/C-2) confirmed that these methyl groups were connected in the C-2 position. The cross-peaks at ẟ 2.42/145.2 (H-12/C-5) and ẟ 2.61/137.2 (H-15/C-7) confirmed that these methyl groups were connected to the C-5 and C-6 positions, respectively (Figure 2). Moreover, a C2 unit was identified at ẟ 3.00/29.14 (H-13/C-13), ẟ 3.75/66.79 (H-14A/C-14), and ẟ 3.56/66.79 (H-14B/C-14). The cross-peaks at ẟ 3.00/145.2 (H-13/C-5) and ẟ 3.00/137.2 (H-13/C-7) confirmed that this unit was attached to the C-6 position of the aromatic ring of compound **14** (Figure 2; Table 1 and Table 2). Furthermore, the 1D ^1^H NMR spectrum and the 1D ^13^C NMR spectrum of **14** showed the presence of a glucosyl substituent and a malonyl substituent, respectively (Figures S1 and S3; Table 1 and Table 2). All the ^1^H and ^13^C resonances belonging to the glucosyl substituent were assigned using the combined information gained from the 1D selective TOCSY spectrum (Figure S2), the 2D ^1^H-^13^C HSQC spectrum (Figure S5), the 2D ^1^H-^13^C H2BC spectrum (Figure S6), and the 2D ^1^H-^1^H COSY spectrum of **14** (Figure S7). The cross-peaks at ẟ 4.20/66.8 (H-1′/C-14), ẟ 3.75/102.9 (H-14A/C-1′), and ẟ 3.56/102.9 (H-14B/C-1′) observed in the 2D ^1^H-^13^C HMBC spectrum of **14** (Figure 3) confirmed that the glucosyl substituent was attached to C-14. The sugar unit was identified as glucose with a *β*-configuration because of the large coupling constant (δ 4.20 d 7.8 Hz; H-1′). The cross-peak at ẟ 5.94/167.2 (H-3/C-1″) observed in the HMBC spectrum (Figure 3) confirmed that the malonyl unit was attached to C-3 (Figure 2).

The stereochemistry of the chiral carbons belonging to C-3 of the 1-indanone core structure was determined via Circular Dichroism (CD) spectroscopy (Figure S9). The CD spectrum of **14** showed a negative Cotton effect at 301 nm and a positive Cotton effect at 333 nm, which was in accordance with 3*R* configuration [9]. Therefore, compound **14** was identified as 3(*R*)-*O*-malonyl pteroside D (Figure 2). A negative molecular ion [M − H]^−^ at *m*/*z* 495.1861 corresponding to C_24_H_32_O_11_ (calculated: *m*/*z* 495.1872; Δ = −2.17 ppm) observed in the high-resolution mass spectrum of compound **14** confirmed this identification (Figure 2 and Figure S8 (in the Supplementary Materials)).

Pterosins are sesquiterpenoids with a 1-indanone core structure; the glucoside version is called a pteroside. This name originates from the fern *Pteridium aquilinum* var. *latiusculum*, which is one of the oldest and most common plants in the world and the first plant source from which such compounds were isolated. At the end of the 19th century, reports of lethal intoxication in cattle were established after consuming *P. aquilinum* [10,11]. Pterosin D and Pteroside D were first isolated by Yoshihira et al. [12] from *P. aquilunum* and have later been found in other fern varieties, such as *P. aqullinum* subsp. *wightianum* (Wall) Shich, *H. punctata* (Thunb) Mett, *J. scammanae* Tryon, *M. speluncae* (L.) Moore, *M. strigosa* (Thunb) Presl, and *D. wilfordii* (Moore) Christ [13]. 

Flavonoids are important natural products of ferns. A multitude of these compounds has hitherto been reported from ferns belonging to Dryopteridaceae, Thelypteridaceae, Selaginellaceae, Equisetaceae, Helminthostachyaceae, Ophioglossaceae, Lygodiaceae, Athyriaceae, and Aspleniaceae species [13]. In the genus Pteridaceae, the flavonoids of the *Pteris* species have been extensively investigated. The main flavonoids of ferns in this compound class are flavones, flavonols, and flavanones [14,15]. All the flavonoids identified in *C. crispa* in this paper are flavonols, where the derivatives of quercetin predominate.

In our ongoing research that aims to identify new lead compounds for future anticancer therapy, the cytotoxic activity of four 1-indanone derivatives (compounds **12**, **13**, **14**, and **15**) were tested towards the acute myeloid leukaemia cell line MOLM-13. Table 3 presents the EC_50_ values after 72 h exposure, which in our previous work, have shown to reveal cytotxic effects at concentrations giving no cell death at 48 h [16].

Moderate and relatively similar cytotoxicity levels towards this cell line were observed for all the compounds, with EC_50_ values ranging from 182.88 ± 0.08 µM for compound **12** to 197.88 ± 0.18 µM for compound **14**. The cytotoxicity of pterosin D and pteroside D could be regarded with the sugar moiety; meanwhile, adding a substituent in the C-3 position and on C-5 decreases the cytotoxicity of these 1-indanone structures [12,17]. 

These compounds exhibited selective cytotoxic activity towards MOLM13, which is illustrated by the fact that none of these compounds were cytotoxic towards the normal cell lines NRK and heart cells, respectively (Table 3). Pterosides are 1-indanone derivatives that are structurally related to ptaquiloside derivatives, which are cancer-promoting agents [18]. However, the cancer-promoting effects are normally not observable within a short timescale of 24 to 72 h, which was applied in our experiments to determine the cytotoxicity of individual compounds. McMorris et al. [19], who studied the structure–activity relationship of the structurally related illudins isolated from *Omphalotus illudens* mushrooms, reported that illudins have a significant anticancer potential. Illudins are structurally related to ptaquiloside structures with fewer cytotoxic effects than ptaquiloside. Some of these compounds, for example, illudin M and illudin S, exhibit significant antibacterial activity [20]. The latter-mentioned compounds are more potent than the pterosides described in this paper, with cytotoxic effects against myeloid leukaemia HL 60 cells in the range of 6-100 nM [21]. According to Liston and Davis [22], EC_50_ concentrations of around 180 µM are within the upper range of biologically relevant anticancer drugs. However, our determinations of cytotoxic activity have been performed on cell cultures; henceforth, the predictions of which concentrations can be achieved in vivo remain elusive.

In comparing the cytotoxicity of the pterosides presented in this work with the other 1-indanone structure, we found that the mild and selective toxicity towards AML MOLM13 cells is a positive discovery worthy of further investigations.

## 3. Materials and Methods

### 3.1. Plant Material

Fresh plant material of *Cryptogramma crispa* was collected during the summers of 2021 and 2022 on the mountain of Fløyen, in Bergen, Norway, at 302 m above sea level (coordinates 60.397687 N and 005.337902 E). A voucher specimen of *C. crispa* was deposited at the herbarium BG, University of Bergen (accession number BG/S-168787). Before extraction, the fresh plant material was stored at −25 °C for preservation.

### 3.2. Extraction and Partition Purification with Organic Solvents

Aerial parts of *Cryptogramma crispa* (1.9 kg) were extracted with 22.5 L HPLC-grade methanol (Sigma-Aldrich, St. Louis, MO, USA) for 72 h maceration at room temperature without mixing. The extraction yield was 8.6% of the wet weight. Considering the water content was 94.1%, the dry weight extraction yield was 46.9%. The methanolic extract was percolated through glass wool and concentrated with rotary evaporators under reduced pressure. The resulting concentrated aqueous extract (700 mL) was purified via liquid–liquid partition three times with petroleum ether (Petroleum ether–ACS reagent, Sigma-Aldrich, Saint Louis, MO, USA) using a total volume of 4.0 L. The resulting water phase was further purified via liquid–liquid partition three times with ethyl acetate (Ethyl Acetate–ACS reagent ≥ 99.5%, Sigma-Aldrich, Saint Louis, MO, USA) using a total volume of 3.2 L. The residual aqueous phase and the ethyl acetate phase were individually concentrated with a rotavapor to a volume of 200 mL each.

### 3.3. XAD-7 Absorption Chromatography

The concentrated residual aqueous extract (200 mL) was added to an Amberlite XAD-7 column (column dimensions 50 × 1000 mm, containing 500 g Amberlite^®^ XAD-7, 20–60 mesh, Sigma-Aldrich, Saint Louis, MO, USA) to remove bulk substances, like sugars, polysaccharides, and free aliphatic amino acids, from the extract when distilled water was used as a mobile phase. Under these solvent conditions, fewer polar and aromatic compounds are absorbed by the XAD-7 column material. The latter compounds are readily eluted from the XAD-7 column when the mobile phase is changed to pure methanol (HPLC grade). The mobile phase gradient consisted of 5.0 L distilled water, followed by 8.0 L methanol. The flow rate was 5 mL/min. This chromatographic separation gave a total of 13 fractions with volumes of 1 L, which were analysed individually via analytical HPLC. The same procedure was conducted with the ethyl acetate phase, whereby 16 fractions were obtained and analysed individually via analytical HPLC.

### 3.4. Sephadex LH-20 Gel Filtration Chromatography

The combined fractions 4–7 and 10–11 from the XAD-7 purification of the water phase were individually concentrated to a volume of 20 mL and further separated individually via gel filtration chromatography with a Sephadex LH-20 column (column dimensions 50 × 1000 mm, containing 500 g of Sephadex^®^ LH-20, Sigma-Aldrich, Saint Louis, MO, USA) using a gradient of super distilled water and methanol containing 0.1% TFA (Trifluoroacetic acid—for HPLC, ≥ 99.0%, Sigma-Aldrich, Saint Louis, MO, USA). The gradient consisted of 2.5 L Water–methanol–TFA 80:20:0.1 *v*/*v*/*v*, followed by 2.5 L Water–methanol–TFA 50:50:0.1 *v*/*v*/*v*, 2.5 L Water–methanol–TFA 30:70:0.1 *v*/*v*/*v*, and finally 2.5 L methanol–TFA 100:0.1 *v*/*v*. The flow rate was 5 mL/min. Each collected fraction had a volume of 90 mL and was analysed via analytical HPLC. From the Sephadex separation of the combined XAD-7 fractions 4–7, 80 fractions were collected. The combined fractions, 12–15, 31–34, 38–39, and 42–48, were afterwards individually separated via preparative HPLC. From the Sephadex separation of the combined XAD-7 fractions 10–11, 30 fractions were collected. The combined fractions 22–24 were thereafter separated via preparative HPLC. A similar procedure was followed for the Sephadex LH-20 separation of the combined XAD-7 fractions 7–10, resulting from the purification of the ethyl acetate partition of the extract. From the Sephadex separation of these combined XAD-7 fractions, 43 fractions were collected. Pure pteroside D (compound **12**) was isolated in fraction 11, while quercetin (compound **1**) and quercetin 3-O-β-galactopyranoside (compound **2**) were identified in fraction 29.

### 3.5. Preparative HPLC

Individual pure compounds of the fractions from Sephadex LH-20 column chromatography were isolated via preparative HPLC (Thermo Scientific preparative HPLC equipped with a Dionex Ultimate 3000 variable wavelength detector) equipped with a C_18_ Ascentis column (column dimensions 250 × 20 mm; 5 µm, spherical particles). A gradient of two solvents was used for elution, consisting of mobile phase A (super distilled water–TFA 99.9:0.1; *v*/*v*) and mobile phase B (acetonitrile–TFA 99.9:0.1; *v*/*v*) (acetonitrile was used for HPLC, gradient grade, ≥99.9%, Sigma-Aldrich, Saint Louis, USA). The elution profile consisted of isocratic elution with A-B (90:10 *v*/*v*) for 4 min, followed by a linear gradient from A-B (90:10 *v*/*v*) to A-B (80:20 *v*/*v*) for the next 10 min, isocratic elution with A-B (80:20 *v*/*v*) for the next 20 min, followed by a linear gradient from A-B (80:20 *v*/*v*) to A-B (70:30 *v*/*v*) for the next 10 min, followed by isocratic elution with A-B (70:30 *v*/*v*) for the next 20 min.

The flow rate was 15 mL/min. Portions of 750 µL were manually injected into the HPLC column and were manually collected based on the peaks that appeared in the online chromatogram recorded at 280 nm. Analytical HPLC was used to analyse the fractions from preparative HPLC separation. Following this strategy, 42.6 mg compound **1**, 66.0 mg of compound **2**, 1.7 mg of compound **3**, 4.6 mg of compound **4**, 2.0 mg of compound **5**, 1.5 mg of compound **6**, 6.5 mg of compound **7**, 12.9 mg of compound **8**, 5.3 mg of compound **9**, 3.9 mg of compound **10**, 21.7 mg of compound **11**, 36.8 mg of compound **12**, 6.3 mg of compound **13**, 2.8 mg of compound **14**, and 18.4 mg of compound **15** were isolated.

### 3.6. Analytical HPLC

Individual samples were analysed using an Agilent Technologies 1260 Infinity II HPLC instrument equipped with a multidiode array detector, an autoinjector, and a 250 × 4.6 mm, 5 μm SUPELCO analytical Ascentis^®^ C18 column. HPLC separation was performed according to the method previously published by Nguyen et al. [23]. Two solvents were used for elution: mobile phase A (super distilled water–TFA 99.9:0.1; *v*/*v*) and mobile phase B (acetonitrile–TFA 99.9:0.1; *v*/*v*), with a flow rate of 1 mL/min, and aliquots of 20 µL were injected. The elution profile began with initial conditions of 90% A and 10% B. Gradient elution followed this for 10 min at 14% B, and then isocratic elution from 10 to 14 min. The subsequent gradient conditions were as follows: 16% B at 18 min, 18% B at 22 min, 23% B at 26 min, 28% B at 31 min, and 40% B at 32 min. This was followed by isocratic elution from 32 to 40 min, gradient elution from 40 to 43 min at 10% B, and final isocratic elution from 43 to 46 min at 10% B [23].

### 3.7. Spectroscopy

High-resolution mass spectra were recorded using a JEOL AccuTOF™ JMS T100LC (JEOL Ltd., Tokyo, Japan) instrument fitted with an electrospray ion source operated in positive mode at a resolving power of approximately 6000 FWHM. Mass spectra were recorded over the mass range of 50–2000 *m*/*z*. The samples were analysed as methanolic solutions and introduced to the ESI spray chamber with weakly acidified (0.01% HCOOH) acetonitrile (Acetonitrile—for HPLC, gradient grade, ≥99.9%, Sigma-Aldrich, Saint Louis, MO, USA) used as a spray reagent.

UV-Vis absorption spectra were recorded online during analytical HPLC analysis over the 210-600 nm wavelength range in steps of 2 nm.

Circular Dichroism (CD) spectra were recorded at 20 °C with a nitrogen atmosphere using a Jasco J-810 spectropolarimeter (Jasco Products LLC, Oklahoma City, OK, USA) equipped with a Peltier temperature control unit. This instrument was used to analyse compound **14** (2.9 mM) dissolved in 100% methanol (methanol for HPLC, ≥99.9%, Sigma-Aldrich, Saint Louis, MO, USA). The spectrum obtained was the average of 6 scans and a buffer scan with 100% methanol, which was subtracted from the spectrum. The spectrum was scanned from 185 to 400 nm. A 1 mm path-length cell was used during analysis.

NMR samples were prepared by dissolving the isolated compound in deuterated dimethylsulfoxide (DMSO-D_6_; 99.96 atom% D, Sigma-Aldrich, Saint Louis, MO, USA). The 1D ^1^H, 1D ^13^CAPT, 2D ^1^H-^13^C HMBC, 2D ^1^H-^13^C HSQC, ^1^H-^13^C HSQC-TOCSY, 2D ^1^H-^13^C H2BC, 2D ^1^H-^1^H COSY, and 2D ^1^H-^1^H ROESY NMR experiments were conducted at 850.1300 MHz and 213.7654 MHz for ^1^H and ^13^C, respectively, using Bruker BioSpin AVANCE III HD 850 MHz instrument (Bruker Biospin AG, Fällanden, Switzerland) equipped with a ^1^H, ^13^C, and ^15^N triple-resonance cryogenic probe at 298 K. The 1D ^1^H NMR experiment (pulse program: zg30) was performed with 32 scans. The sweep width was 16 ppm, and the acquisition time was 1 min 57 s. The 1D ^1^H selective TOCSY NMR experiment (pulse program: seldigpzs) was performed with 16 to 128 scans, depending on the individual sample concentrations. The sweep width was 16 ppm, and the acquisition time was between 1 min 57 s (NS = 16) and 10 min 19 s (NS = 128). The 1D ^13^CAPT NMR experiment (pulse program: jmod) was performed with 512 to 15 800 scans, depending on the individual sample concentrations. The sweep width was 240 ppm, and the acquisition time was between 23 min 7 s (NS = 512) and 11 h 48 min 18 s (NS = 15 800). The 2D ^1^H-^13^C HMBC NMR experiment (pulse program: hmbcgplpndqf) was performed with 2 to 24 scans, depending on the individual sample concentrations and 256 experiments in F1. The sweep widths were 10 to 14 ppm for ^1^H and 220 ppm for ^13^C. The acquisition time was between 15 min 55 s (NS = 2) and 3 h 5 min 14 s (NS = 24). The 2D edited ^1^H-^13^C HSQC experiment (pulse program: hsqcedetgpsisp2.3) was performed with 2 to 8 scans, depending on the individual sample concentrations, and 256 experiments in F1. The sweep widths were 10 ppm for ^1^H and 160 ppm for ^13^C. The acquisition time was between 20 min 1 s (NS = 2) and 1 h 18 min 8 s (NS = 8). The 2D ^1^H-^13^C H2BC NMR experiment (pulse program: h2bcetgpl3) was performed with 2 to 16 scans, depending on the individual sample concentrations and 256 experiments in F1. The sweep widths were 10 ppm for ^1^H and 160 ppm for ^13^C. The acquisition time was between 15 min 57 s (NS = 2) and 2 h 4 min 13 s (NS = 16). The 2D ^1^H-^13^C HSQC-TOCSY NMR experiment (pulse program: hsqcdietgpsisp2) was performed with 2 to 16 scans, depending on the individual sample concentrations and 256 experiments in F1. The sweep widths were 10 ppm for ^1^H and 160 ppm for ^13^C. The acquisition time was between 21 min 11 s (NS = 2) and 2 h 44 min 42 s (NS = 16). The 2D ^1^H-^1^H ROESY NMR experiment (pulse program: roesyphpp2) was performed with 8 scans and 256 experiments in F1. The sweep width was 10 ppm. The acquisition time was 1 h 25 min 36 s. The 2D ^1^H-^1^H COSY NMR experiment (pulse program: cosygpppqf) was performed with 8 scans and 256 experiments in F1. The sweep width was 10 ppm. The acquisition time was 1 h 25 min 36 s.

### 3.8. Cytotoxicity

Stock solutions were prepared by dissolving pure compounds to a final concentration of 20 mM in DMSO. The normal rat kidney epithelial cells (NRK, ATCC no.: CRL-6509) and rat cardiomyoblasts (H9c2, ATCC no.: CRL-1446) were cultured in DMEM medium supplemented with 10% foetal bovine serum (FBS, Invitrogen, Carlsbad, CA, USA). When the cells reached 80% confluence, they were detached in a mild trypsin treatment (0.33 mg/mL trypsin for 5 min at 37 °C), centrifuged (160× *g*, 4 min), and reseeded in fresh medium to 25% confluence.

The AML cell line MOLM13 (DSMZ no.: ACC554 [24]) was cultured in RPMI-1640 medium enriched with 10% FBS 8mM L-glutamine (Sigma Life Science, London, UK). The cells were kept in suspension cultures at a density of between 150,000 and 700,000 cells/mL.

All media were supplemented with 1 IU/mL penicillin and 1 mg/mL streptomycin (both from Cambrex, Liège, Belgium) and incubated in a humidified atmosphere (37 °C, 5% CO_2_).

For the cytotoxicity experiments, the NRK and H9c2 cells were seeded in 96-well tissue culture plates (4000 cells/well, 0.1 mL) and left overnight to attach before adding the compounds. The MOLM13 cells were seeded in 96-well tissue culture plates at 20,000 cells/well in 0.1 mL on the day of the experiment. 

The compounds dissolved in DMSO were added to the cells, and the plates were kept overnight before adding the tetrazolium salt WST-1 according to the manufacturer’s instructions (Roche Diagnostics GmbH, Mannheim, Germany). The plates were further incubated for two hours before the signal was recorded at 450 nm with a reference at 620 nm. For blank subtraction, only the medium-added WST-1 and plant compounds were used. This procedure was conducted after 24 and 72 h.

After the recording of WST-1, the cells were then fixed with 2% buffered formaldehyde (pH 7.4) with 0.01 mg/mL of the DNA-specific fluorescent dye, Hoechst 33342. The presence of dead (apoptotic or necrotic) cells was verified via differential interference contrast and fluorescence microscopy as previously described by Oftedal et al. and Myhren et al. [25,26]. 

EC_50_ values were determined using four-parameter regression analysis described by Viktorsson et al. [27] using SigmaPlot v14 software (Systat Software Inc., San Jose, CA, USA).

## 4. Conclusions

For the first time, 15 natural products, including the previously undescribed compound 3(*R*)-*O*-malonyl pteroside D, have been characterised from *Cryptogramma crispa* (L.) R. Br. The four isolated 1-indanone derivatives were tested for their cytotoxic activity towards the acute myeloid leukaemia cell line MOLM-13, and all these compounds exhibited moderate and relatively similar cytotoxicity. This is particularly noteworthy considering that pterosides are 1-indanone derivatives of plants that are structurally related to ptaquiloside compounds, which are known their cancer-promoting agents.

The indane core, a common feature in these compounds, appears to be significant. Illudins, natural products with structures related to ptaquiloside, have been reported to have significant anticancer potential and fewer cytotoxic effects than ptaquiloside. 

This study provides valuable insights into the potential of 1-indanone derivatives as lead compounds for future anticancer therapy. Their selective cytotoxic activity towards the MOLM13 cell line and lack of cytotoxicity towards normal cell lines highlight their potential for targeted cancer treatment. Moreover, future structure–activity relationship studies could reveal the modifications that improve the active compounds’ potency and possibly selectivity. As such, we believe that our active compounds merit further investigations to reveal their potential as possible drug leads.

## Figures and Tables

**Table 1:** ^1^H chemical shift values (ppm) and coupling constants (Hz) of Pteroside D (**12**), Pteroside X (**13**), 3-malonyl-pteroside D (**14**), and Pterosin D (**15**) dissolved in DMSO-D_6_ at 298K.

	Compound 12	Compound 13	Compound 14	Compound 15
	δ ^1^H	δ ^1^H	δ ^1^H	δ ^1^H
1				
2				
3	4.64	4.66	5.94 s	4.64
4	7.30 s	7.62 s	7.28 s	7.30 s
5				
6				
7				
8				
9				
10	1.08 s	1.10 s	1.19 s	1.08 s
11	0.91 s	0.92	0.96 s	0.91 s
12	2.41 s	4.65 s	2.42 s	2.40 s
13	2.97 m	2.95 t 7.9	3.00 m	2.84 dd 8.5, 7.3
14A	3.75 m	3.75 m	3.75 m	3.45 dd 8.5, 7.3
14B	3.55 m	3.54 dt 10.1, 7.9	3.56 m	
15	2.57	2.58 s	2.61 s	2.56 s
14-*O*-*β*-glc				
1′	4.20 d 7.8	4.18 d 7.8	4.20 d 7.8	
2′	2.98 dd 2.8; 9.1	2.95 dd 9.0, 7.8	2.94 dd 9.0, 7.8	
3′	3.15 dd 8.5; 9.1	3.12 t 8.9	3.12 t 8.9	
4′	3.06 dd 8.5; 9.7	3.03 dd 9.7, 8.8	3.02 dd 9.8, 8.8	
5′	3.09 ddd 2.2; 5.8; 9.7	3.08 ddd 9.7, 6.0, 2.2	3.08 ddd 9.8, 6.0, 2.2	
6A′	3.66 dd 2.2; 11.8	3.64 dd 11.8, 2.2	3.64 dd 11.8, 2.2	
6B′	3.44 dd 5.8; 11.8	3.42 dd 11.8, 6.0	3.41 dd 11.8, 6.0	
3-*O*-malonyl				
1″				
2″			3.50 s	
3″				

**Table 2:** ^13^C chemical shift values (ppm) of Pteroside D (**12**), Pteroside X (**13**), 3-malonyl-pteroside D (**14**), and Pterosin D (**15**) dissolved in DMSO-D_6_ at 298K.

	Compound 12	Compound 13	Compound 14	Compound 15
	δ ^13^C	δ ^13^C	δ ^13^C	δ ^13^C
1	209.26	209.23	207.21	209.12
2	51.06	51.01	49.77	50.88
3	75.45	75.35	77.61	75.25
4	125.18	121.75	125.42	124.92
5	144.48	148.08	145.19	144.20
6	136.60	134.93	138.15	137.19
7	136.55	136.25	137.15	136.15
8	129.62	129.88	129.91	129.39
9	153.19	153.12	147.74	152.81
10	23.04	22.85	23.46	22.95
11	20.67	20.64	20.11	20.59
12	21.03	61.38	20.96	20.95
13	29.22	28.10	29.14	32.22
14A	67.13	67.43	66.79	59.81
14B				
15	13.58	13.22	13.4	13.50
14-*O*-*β*-glc				
1′	103.08	102.95	102.91	
2′	73.73	73.56	73.56	
3′	77.07	76.87	76.90	
4′	70.33	70.15	70.19	
5′	77.10	76.99	77.02	
6A′	61.31	61.14	61.16	
6B′				
3-*O*-malonyl				
1″			167.18	
2″			41.62	
3″			168.00	

**Table 3:** Cytotoxicity of compounds **12**–**15** against three mammalian cell lines. The compounds were dissolved in DMSO. The cells were tested for metabolic activity after 72 h of incubation. The EC_50_ values were determined via non-linear regression from 3 independent experiments (MOLM13), as described in the methods section. The data from H9c2 and NRK cells originate from three experiments. “-” denotes that no data are available due to low or no observed toxicity above concentrations of 200 µM.

	MOLM13 (µM)	H9c2 (µM)	NRK (µM)
Compound **12**	182.88 ± 0.08	-	-
Compound **13**	191.66 ± 0.13	-	-
Compound **14**	197.88 ± 0.18	-	-
Compound **15**	189.96 ± 0.16	-	-

## Data Availability

Data are contained within the article and Supplementary Materials.

## Supplementary Materials

The following supporting information can be downloaded at: https://www.mdpi.com/article/10.3390/molecules28237723/s1, Figure S1: 1D ^1^H NMR spectrum of 3-manolyl pterosideD; Figure S2: 1D ^1^H selective TOCSY NMR spectrum of 3 manolyl pteroside D; Figure S3: 1D ^13^CAPT NMR spectrum of 3 manolyl pteroside D; Figure S4: 2D ^1^H-^13^C HMBC NMR spectrum of 3 manolyl pteroside D; Figure S5: 2D ^1^H-^13^C HSQC NMR spectrum of 3 manolyl pteroside D; Figure S6: 2D ^1^H-^13^C H2BC NMR spectrum of 3 manolyl pteroside D; Figure S7: 2D ^1^H-^1^H COSY NMR spectrum of 3 manolyl pteroside D; Figure S8: High Resolution Mass Spectrum of 3 manolyl pteroside D; Figure S9: Circular Dichroism (CD) spectrum of 3 manolyl pteroside D; Figure S10: UV spectrum of 3 manolyl pteroside D.
