# Supplementary material for: Cytotoxic Natural Products Isolated from Cryptogramma crispa (L.) R. Br
Source: Molecules. 2023 Nov 23;28(23):7723. doi: 10.3390/molecules28237723 (PMC10708030; doi:10.3390/molecules28237723)

Cytotoxic Natural Products Isolated from *Cryptogramma crispa* (L.) R. Br.

Supplementary Data

NMR spectra

Mass spectrum

CD spectrum

UV spectrum

Figure S1. 1D ^{1}H NMR spectrum of 3-manolyl pteroside D

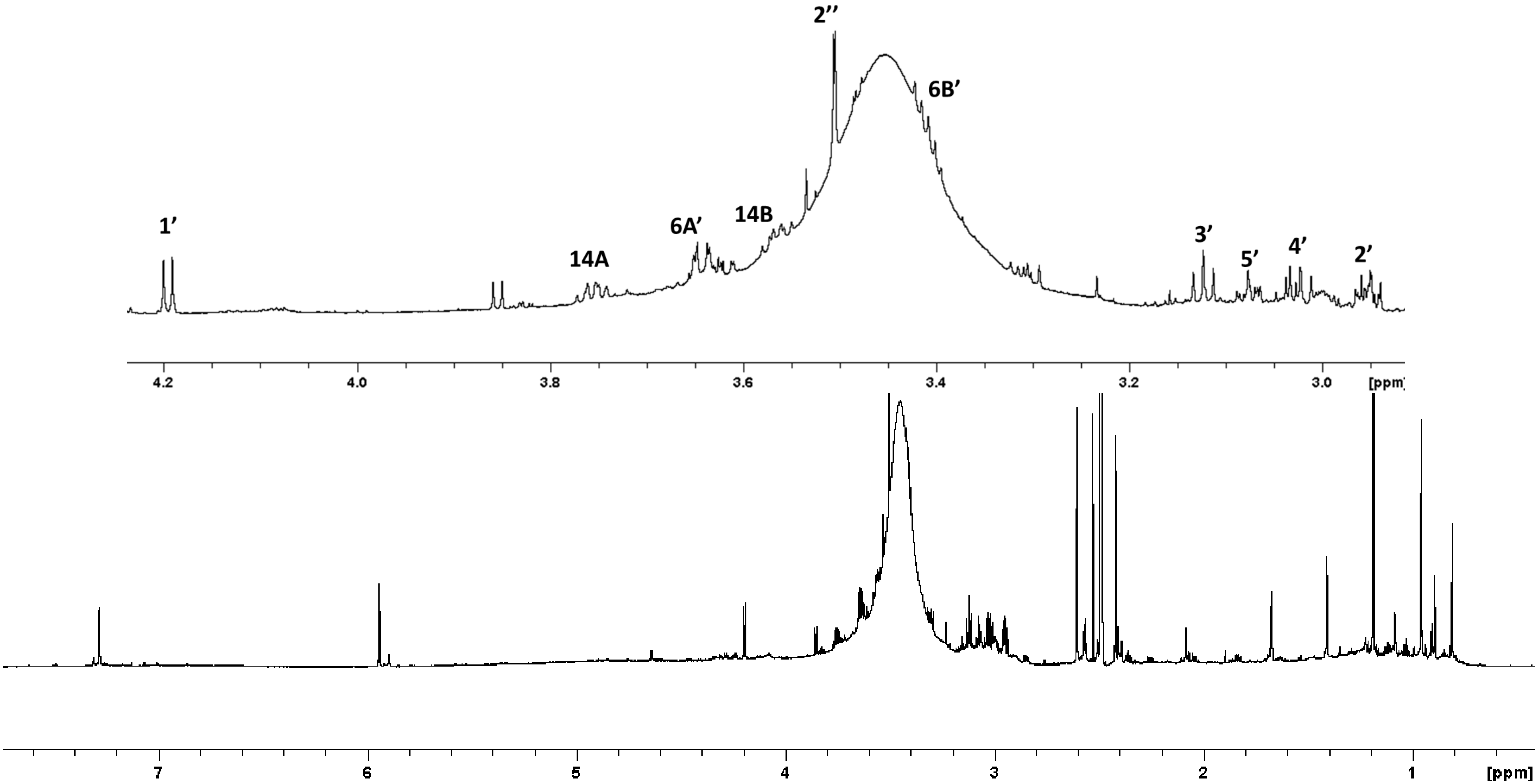

Figure S2. 1D ^{1}H selective TOCSY NMR spectrum of 3-manolyl pteroside D

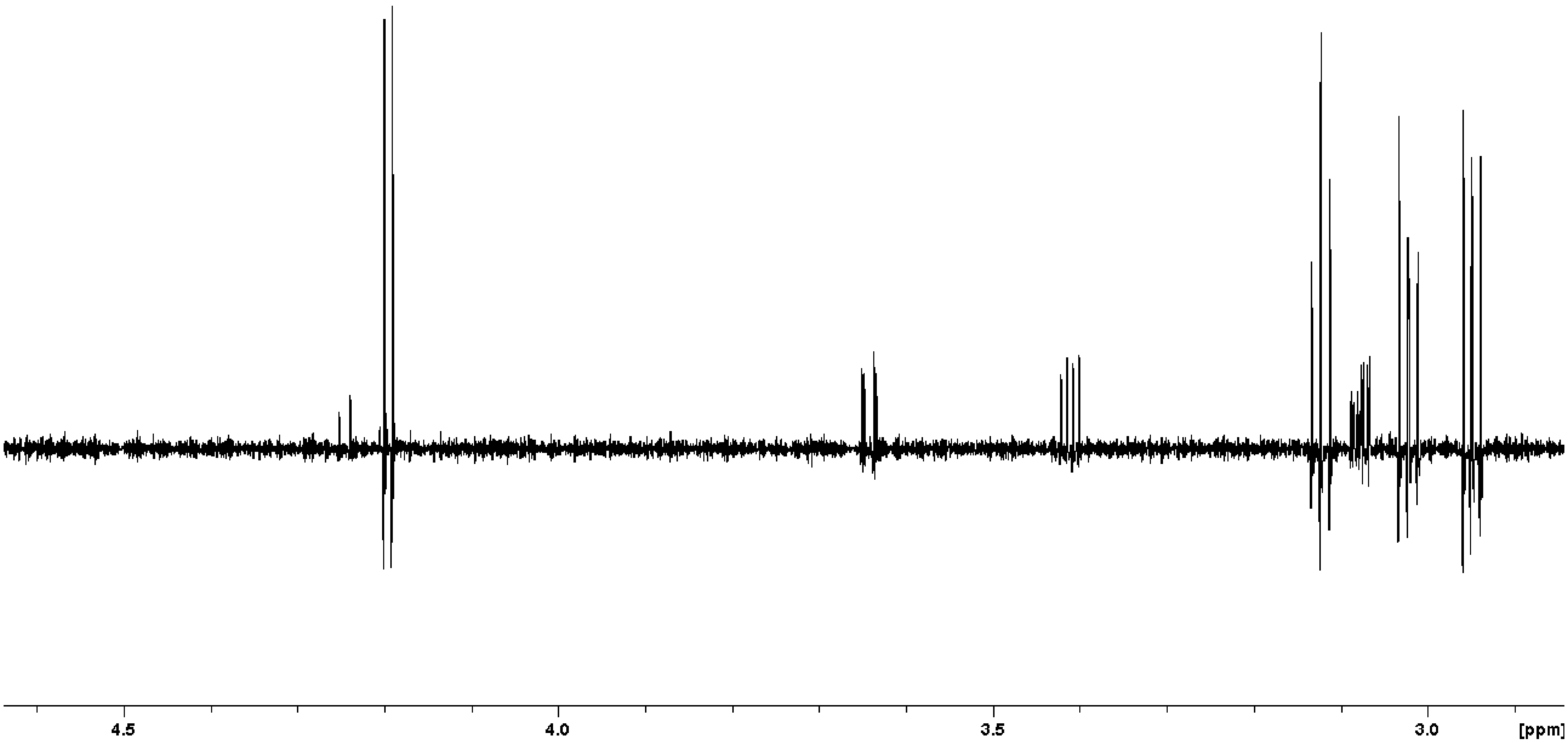

Figure S3. 1D ^{13}CAPT NMR spectrum of 3-manolyl pteroside D

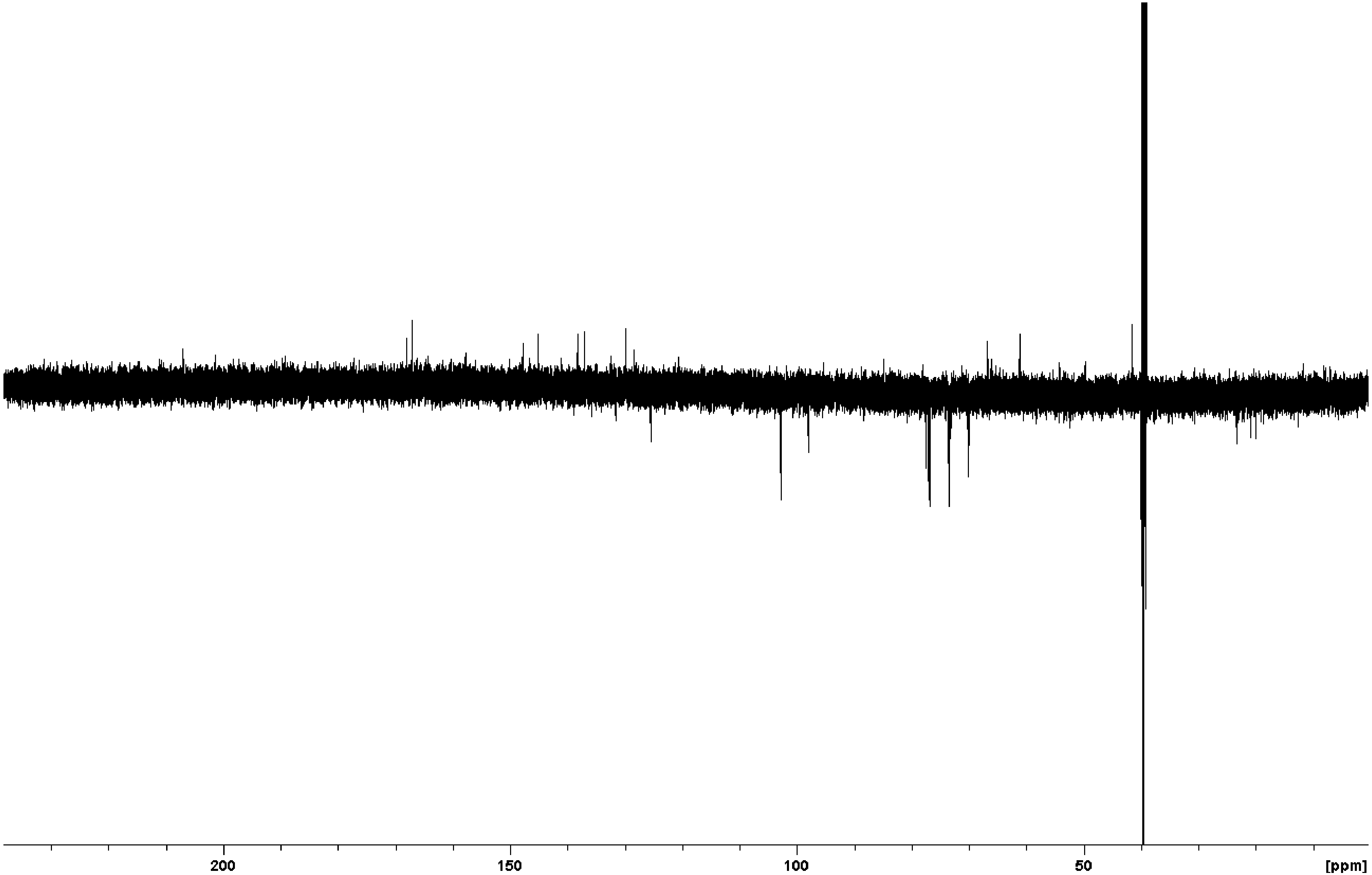

Figure S4. 2D ^{1}H-^{13}C HMBC NMR spectrum of 3-manolyl pteroside D

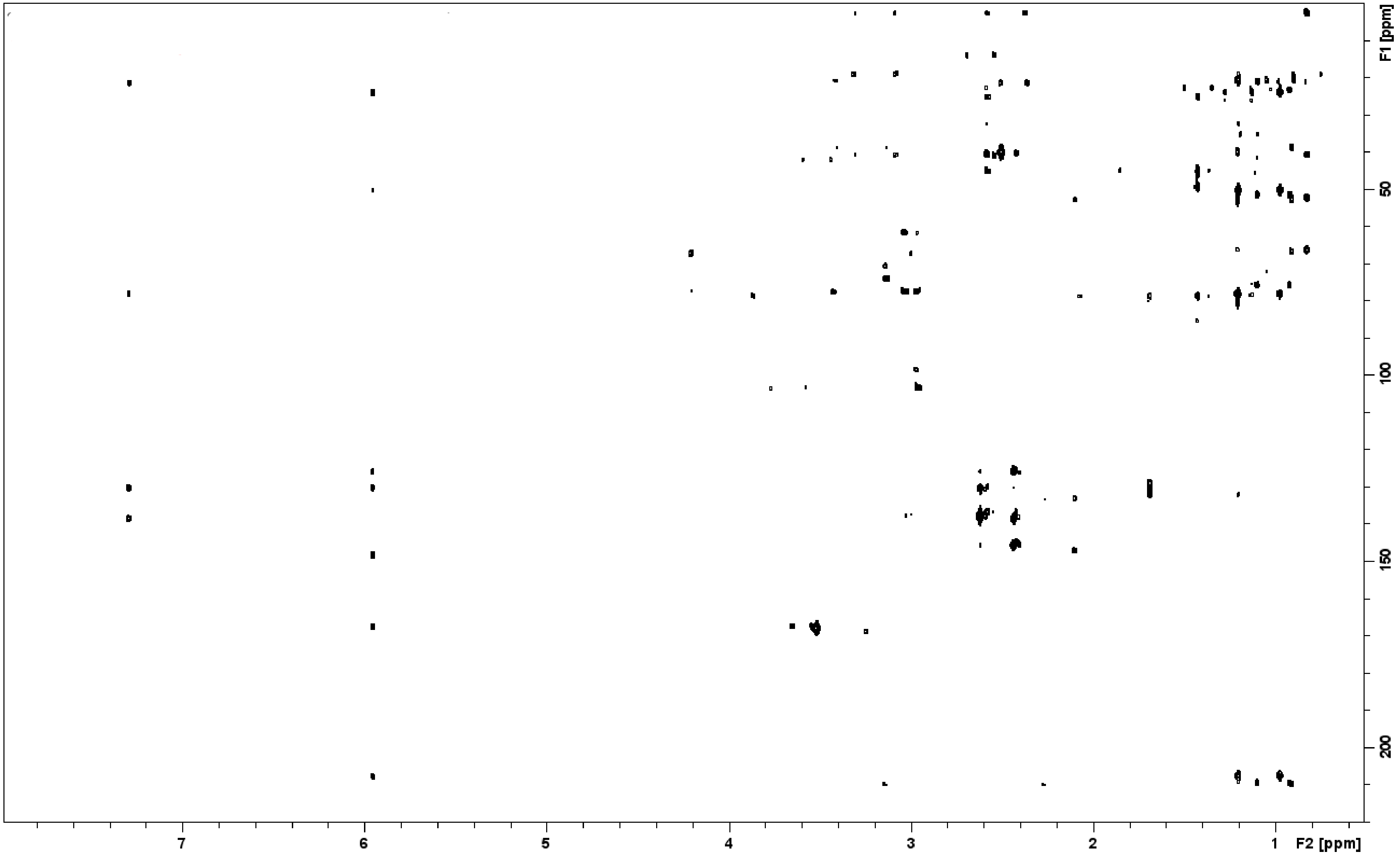

Figure S5. 2D ^{1}H-^{13}C HSQC NMR spectrum of 3-manolyl pteroside D.

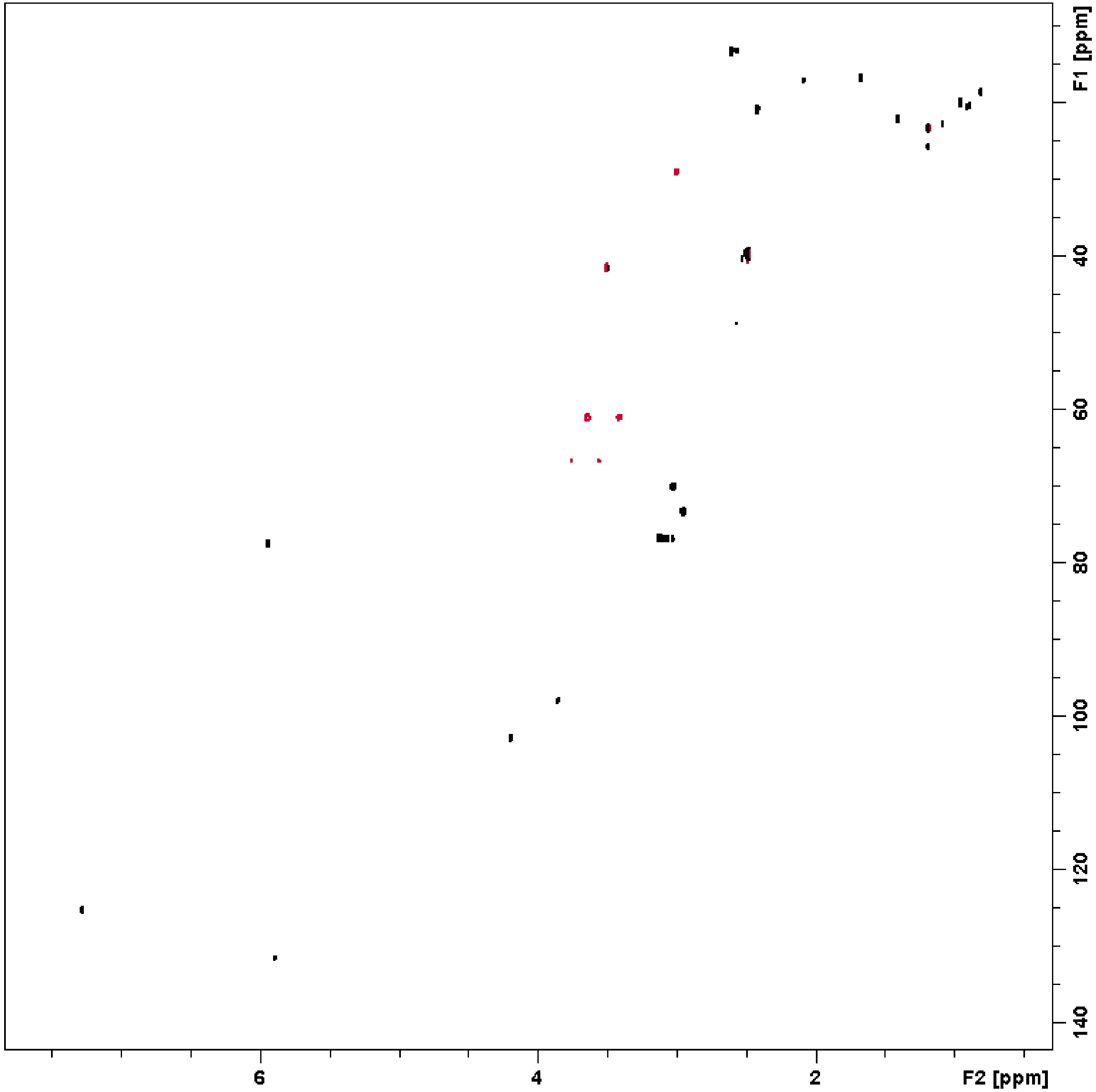

Figure S6. 2D ^1H-^{13}C H2BC NMR spectrum of 3-manolyl pteroside D.

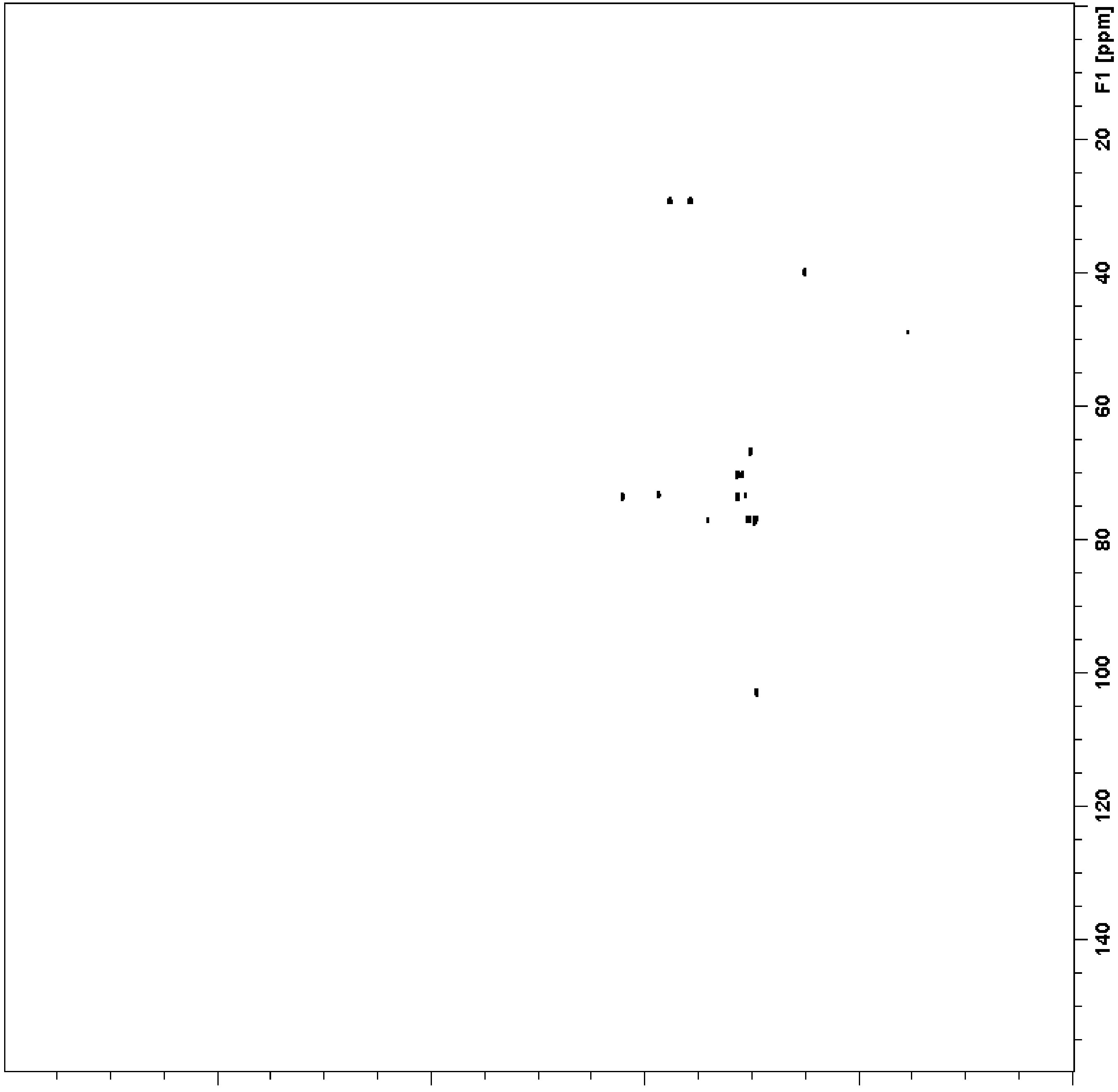

Figure S7. 2D ^{1}H-^{1}H COSY NMR spectrum of 3-manolyl pteroside D.

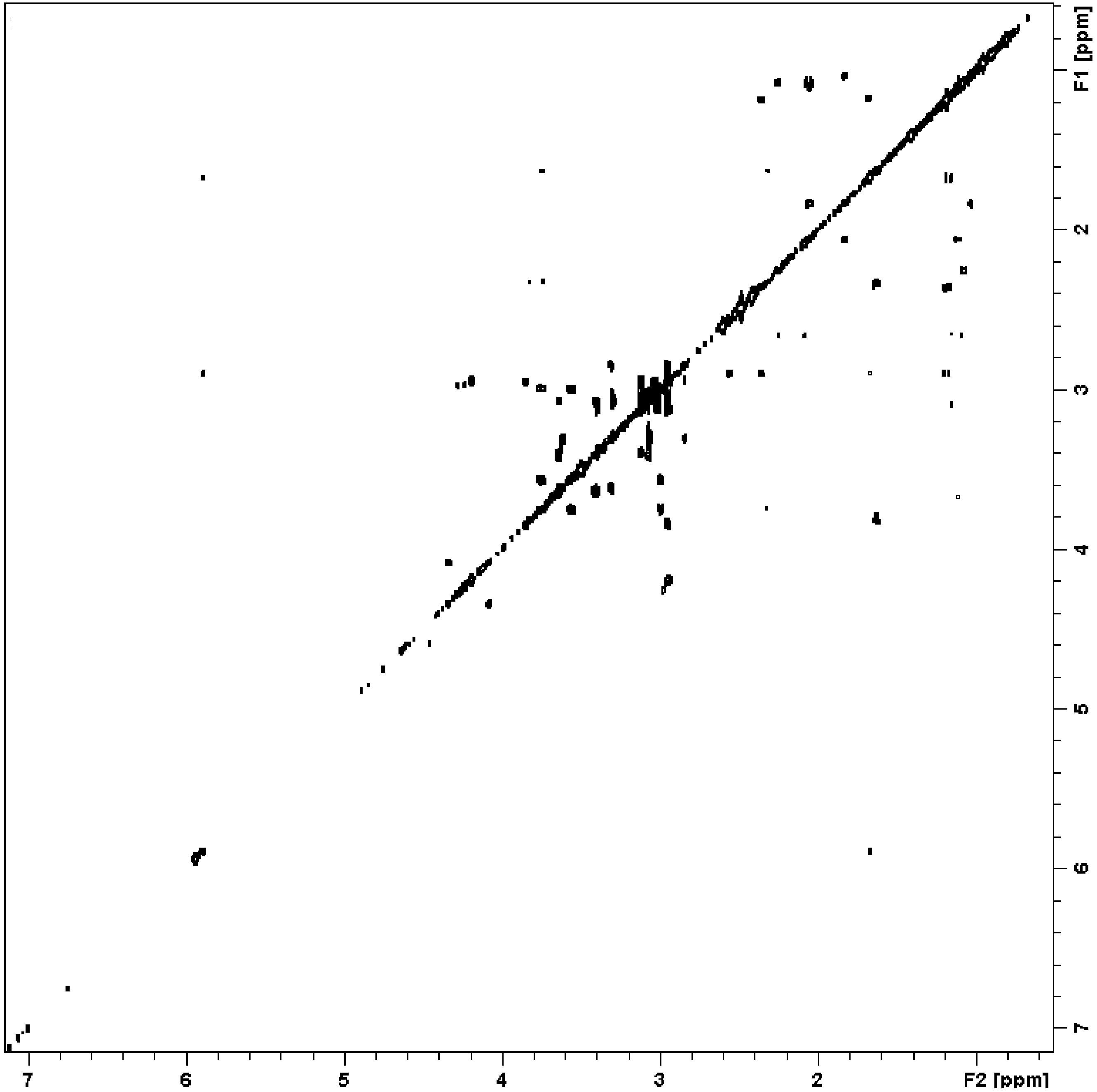

Figure S8. High Resolution Mass Spectrum of 3-manolyl pteroside D.

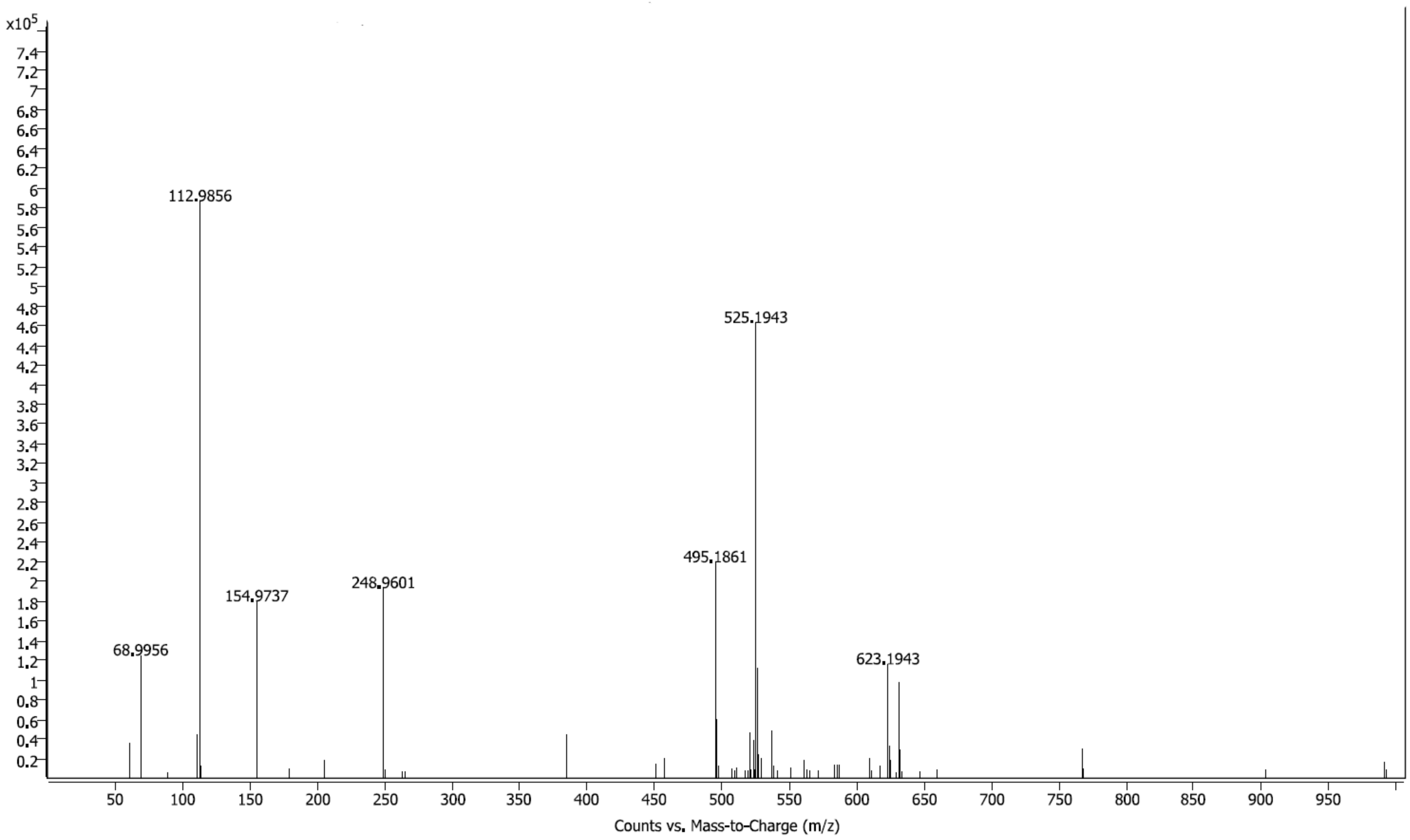

Figure S9. Circular Dichroism (CD) spectrum of 3-manolyl pteroside D.

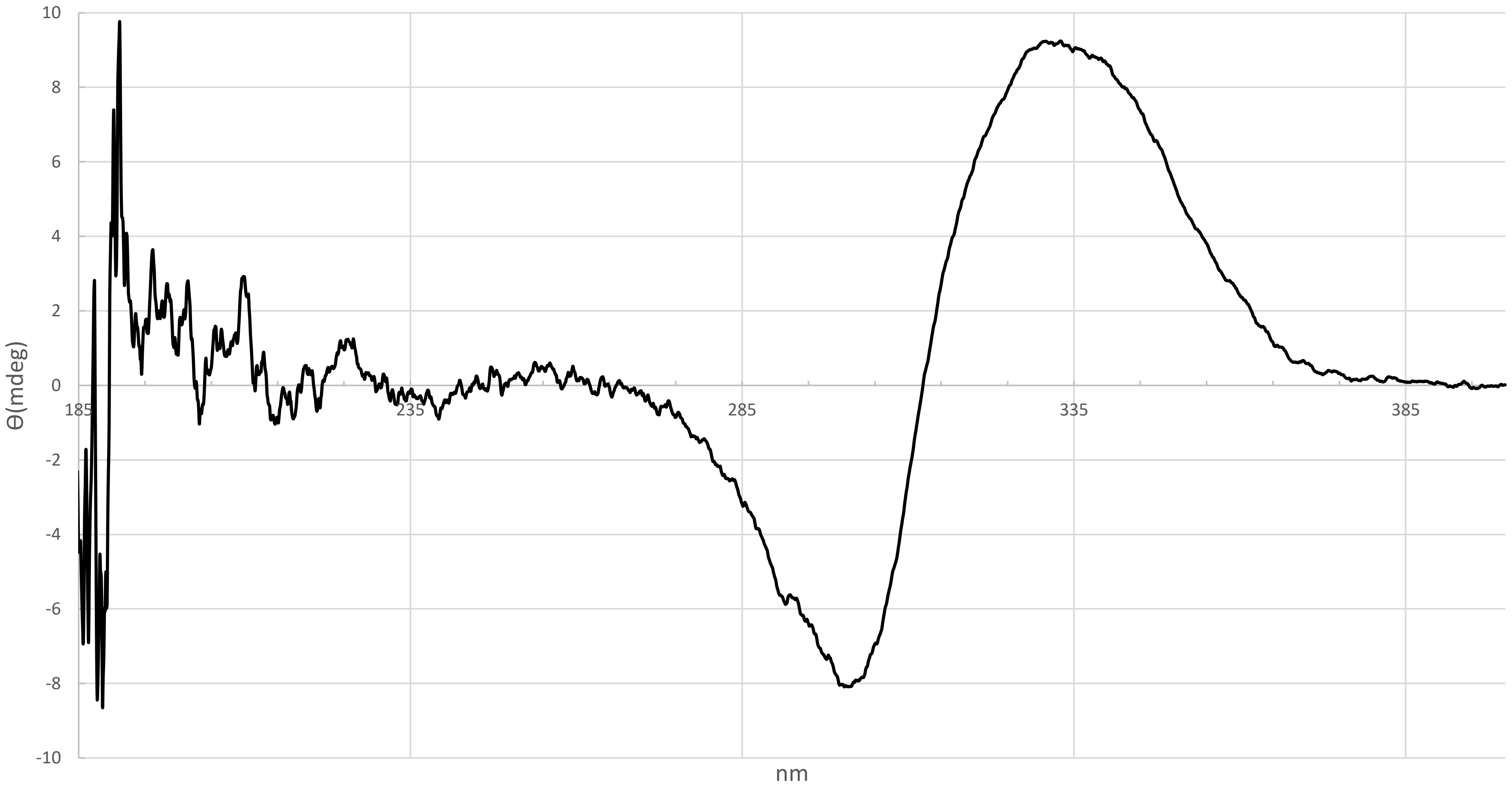

Figure S10. UV spectrum of 3-manolyl pteroside D.

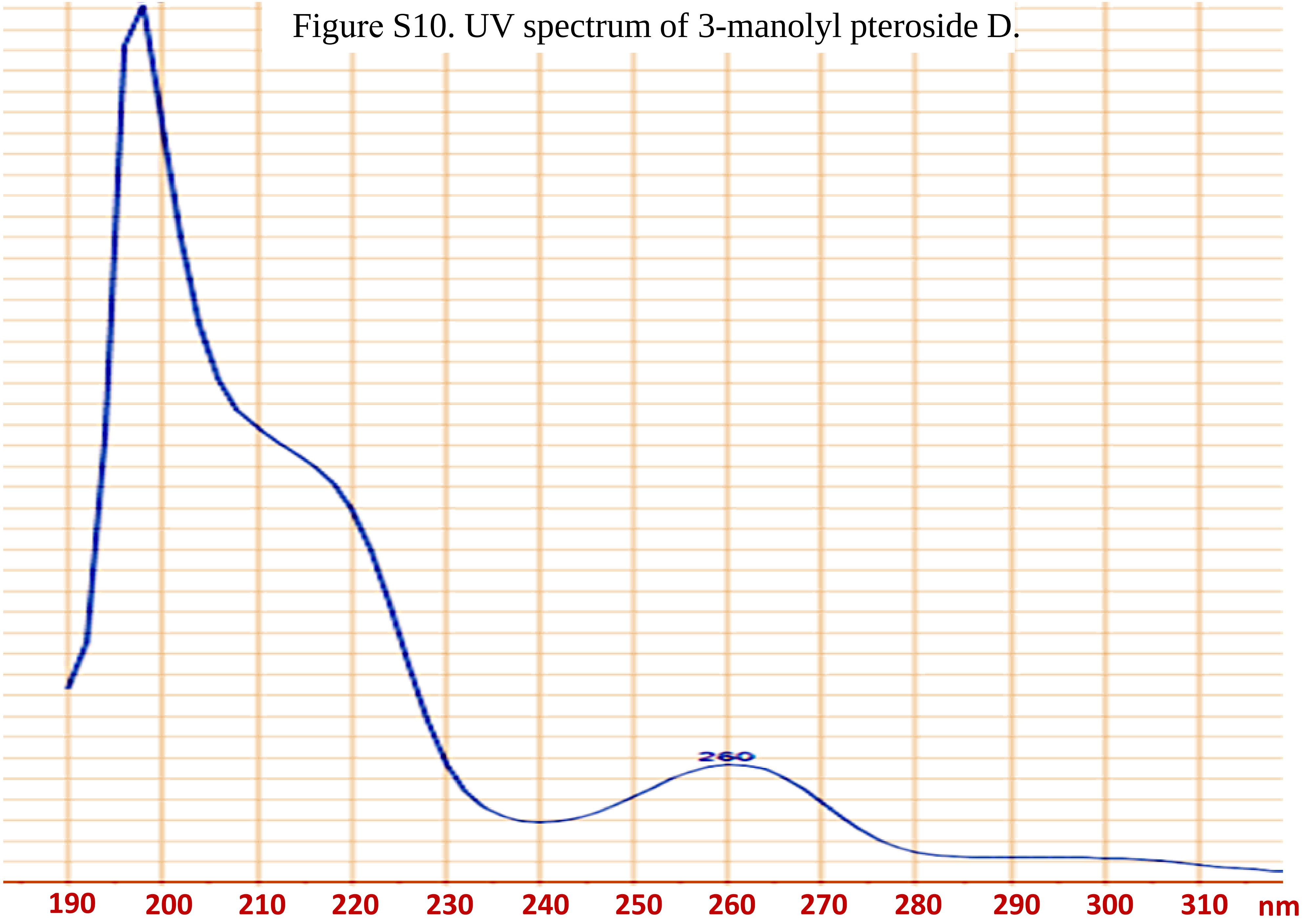

Supplement: Supplementary file 1 [file molecules-28-07723-s001.zip › molecules-2720897-supplementary.pdf]
